# Notes and News

**Published:** 1899-10-28

**Authors:** 


### Notes and News.

(Collected and Communicated.)

HOSPITAL MEETINGS.

Hospital Saturday Fund.—Annual Meeting at half-past three, on Saturday, October 28th, at the Mansion House.
Victoria Hospital for Children.—Festival Dinner at eight p.m., on November 8th, at the Hotel Cecil, Field Marshal Lord Roberts in the chair.

Dr. James Dunsmure has been elected President of the Royal College of Surgeons, Edinburgh, for the ensuing year.

The employés of the General Post Office have contributed £207 to the Prince of Wales's Fund during the present year.

At the meeting of the London County Council last Tuesday the report of the committee recommending the clearance of four insanitary areas in different parts of London, the expenditure for which amount to between £500,000 and £600,000, was passed.

On the occasion of the Harveian Oration at the Royal College of Physicians the striking colour scheme presented by the doctors' gowns and hoods was variegated by the handsome costume of the Chinese Ambassador, who sat at the president's right hand.

The new Redhead wing at the Ingham Infirmary, South Shields, has been opened by Mr. Robson, Q.C. The new wing contains two main wards and four isolation wards, day-rooms, nurses' kitchen, and sitting-room. Accommodation is provided for 48 more patients. The building has cost about £8,000.

At the half-yearly court of governors of the Royal Orthopædic Hospital, Mr. H. H. Marks, M.P., being in the chair, it was stated that the hospital, which had been closed to in-patients since midsummer, would shortly be reopened. Nearly £2,000 had been expended in reconstruction of the drainage, and other sanitary improvements.

The Sunday Concert Society, which has held Sunday concerts for some time at the Queen's Hall, was obliged to announce at a general meeting that the receipts had fallen short of expenses. It was originally intended that any surplus should be devoted to the Prince of Wales's Fund, but, no surplus existing, the Fund has not benefited. All the same, the concerts will be continued, and Mr. Newman, the lessee of the hall, is making up the deficits on the present occasion.

It is stated that the American troops on the transports coming from Manila were very badly accommodated, and that the sick were especial sufferers. A large amount of dysentery prevailed.

Dr. W. J. Potts, Assistant Medical Officer of the Brook Hospital, Shooter's Hill, has been elected Medical Superintendent of the New Bethnal Green Infirmary. The position carries with it a salary of £500 per annum, house, and lighting.

The Marquis of Bristol has just opened the new Victoria wing of the East Suffolk and Ipswich Hospital, which is the Jubilee memorial for the neighbourhood. The building consists of day room for men, servants' hall, two wards for 14 beds each, with usual offices. At the opening ceremony an anonymous donor made a gift of £100 to improve the operating theatre at the hospital.

In a very carefully worked out article in the *Charity Organisation Review*, signed by W. Chance, it is shown that, while in certain selected unions there has of late years been a general reduction of pauperism, this reduction has been still more marked among those above 65 years of age than it has among those between 16 and 65; and the conclusion is arrived at that old age pauperism is decreasing, and that it should continue to decrease under our existing Poor Law. Finally, he asks, if the figures from other unions show that old age pauperism has been decreasing as fast as his figures go to prove, "might not the country do well to leave the question of the establishment of any system of old age pensions alone for a time?"

Dublin is about to provide further hospital accommodation for its infectious cases, which appears to be a very necessary step. But there is also a cry that the general hospital accommodation is not sufficient or well distributed, and it has been suggested in the local press that a hospital census should be taken showing the amount of accommodation provided in each hospital, and how it has been occupied, from what localities, and whether the patients have been received gratuitously or for payment. The suggestion is a very sensible one, but whether the institutions will assist in placing themselves in line with one another in such a manner as to clearly show the public where overlapping or want of relief is felt is another matter.

Seldom has the occupant of the historic pulpit of St. Paul's gazed on a more picturesque scene than that which met the eyes of the Bishop of Stepney when he stood up last Thursday evening to preach to the Guild of St. Luke. The gorgeous habiliments of the Lord Mayor and Sheriffs, the brilliant gowns of the doctors of both sexes—the ladies wearing their college caps—and the contrast presented by the every-day attire of the general public, would have impressed a less observant man than Dr. Winnington Ingram. If he wanted inspiration he must have caught it from such a representative congregation, and his sermon on the relations of Religion and Medicine was as lofty in tone as it was telling in delivery. There was a deep silence as, in his closing words, he paid a glowing tribute to the memory of Bishop Hicks of Bloemfontein, "the only Fellow of the College of Physicians in Holy Orders."

